# Chemically induced hypoxia by dimethyloxalylglycine (DMOG)-loaded nanoporous silica nanoparticles supports endothelial tube formation by sustained VEGF release from adipose tissue-derived stem cells

**DOI:** 10.1093/rb/rbab039

**Published:** 2021-08-14

**Authors:** Sarah Zippusch, Karen F W Besecke, Florian Helms, Melanie Klingenberg, Anne Lyons, Peter Behrens, Axel Haverich, Mathias Wilhelmi, Nina Ehlert, Ulrike Böer

**Affiliations:** 1 Lower Saxony Centre for Biomedical Engineering, Implant Research and Development (NIFE), Hannover Medical School, Stadtfelddamm 34, 30625 Hannover, Germany; 2 Division for Cardiac, Thoracic-, Transplantation- and Vascular Surgery, Hannover Medical School, Carl-Neuberg-Straße 1, 30625 Hannover, Germany; 3 Institute of Inorganic Chemistry, Leibniz University Hannover, Callinstraße 9, 30167 Hannover, Germany; 4 Cluster of Excellence Hearing4all, Carl-von-Ossietzky-Straße 9-11, 26129 Oldenburg, Germany; 5 Department of Vascular- and Endovascular Surgery, St. Bernward Hospital, Treibestraße 9, 31134 Hildesheim, Germany

**Keywords:** pre-vascularization, tissue engineering, adipose tissue-derived stem cells, dimethyloxalylglycine, nanoporous silica nanoparticles

## Abstract

Inadequate vascularization leading to insufficient oxygen and nutrient supply in deeper layers of bioartificial tissues remains a limitation in current tissue engineering approaches to which pre-vascularization offers a promising solution. Hypoxia triggering pre-vascularization by enhanced vascular endothelial growth factor (VEGF) expression can be induced chemically by dimethyloxalylglycine (DMOG). Nanoporous silica nanoparticles (NPSNPs, or mesoporous silica nanoparticles, MSNs) enable sustained delivery of molecules and potentially release DMOG allowing a durable capillarization of a construct. Here we evaluated the effects of soluble DMOG and DMOG-loaded NPSNPs on VEGF secretion of adipose tissue-derived stem cells (ASC) and on tube formation by human umbilical vein endothelial cells (HUVEC)-ASC co-cultures. Repeated doses of 100 µM and 500 µM soluble DMOG on ASC resulted in 3- to 7-fold increased VEGF levels on day 9 (*P* < 0.0001). Same doses of DMOG-NPSNPs enhanced VEGF secretion 7.7-fold (*P* < 0.0001) which could be maintained until day 12 with 500 µM DMOG-NPSNPs. In fibrin-based tube formation assays, 100 µM DMOG-NPSNPs had inhibitory effects whereas 50 µM significantly increased tube length, area and number of junctions transiently for 4 days. Thus, DMOG-NPSNPs supported endothelial tube formation by upregulated VEGF secretion from ASC and thus display a promising tool for pre-vascularization of tissue-engineered constructs. Further studies will evaluate their effect in hydrogels under perfusion.

## Introduction

While the field of tissue engineering has emerged as one of the most promising alternatives to organ or tissue transplantation, there are still obstacles that have yet to be overcome, with the major issue being proper vascularization of larger three-dimensional (3D) tissue constructs in order to guarantee implant survival. Passive diffusion of nutrients and oxygen in tissue constructs is limited to a range of 150–200 µm, which raises the need for an extensive vascular network within bioartificial tissue constructs to supply incorporated cells appropriately [1].

Vascularization of a tissue construct involves two mechanisms, (i) vasculogenesis, which is the formation of new blood vessels from progenitor cells and (ii) angiogenesis, which is the sprouting of blood vessels from already existing vessels. These mechanisms can be influenced by several factors, one of those being hypoxia, which is known to play a pathophysiological role in many diseases, such as atherosclerosis or cancer [2]. Tissue hypoxia is induced by an imbalance between oxygen demand and supply and is considered to be the key player in the upregulation of angiogenesis by enhancing the expression of vascular endothelial growth factor (VEGF). VEGF is the most important pro-angiogenic growth factor needed in the first stages of blood vessel sprouting [3]. Hypoxia-inducible factors (HIF-1, HIF-2, HIF-3) and their oxygen-regulated subunits HIF-1α and HIF-1β are considered to be the mean mediators of hypoxia, since they upregulate VEGF expression as transcription factors and can thereby regulate the process of angiogenesis [4]. Under normoxic conditions, prolyl hydroxylases (PHDs) use oxygen and α-ketoglutarate (α-KG) to promote the degradation of HIFs. However, with oxygen levels decreasing, HIFs are no longer hydrolysed, leading to HIF-1α and HIF-1β dimerization and target gene upregulation [3, 5]. Apart from endothelial cells (EC), adipose tissue-derived stem cells (ASC) are key players in blood vessel formation. ASC have been shown to stabilize and regulate EC in the process of capillarization and wound healing [6, 7] by direct cell to cell interactions as well as secretion of numerous angiogenic growth factors, such as platelet-derived growth factor (PDGF) and VEGF.

Although an ubiquitous hypoxic state within and around tissue constructs can be achieved rather easily *in vitro* by utilizing a hypoxic incubation chamber, it is fairly difficult to tune and more importantly direct hypoxic conditions within different areas of the tissue construct. Thus, incubator-controlled hypoxia is not always suitable for tissue engineering applications. Consequently, a more precise technique to use the beneficial effects of hypoxia is needed. For this purpose, dimethyloxalylglycine (DMOG) is a promising candidate. DMOG is a di-esterified prodrug of N-oxalylglycine (NOG), which is an active hypoxia-inducing agent. DMOG is cell-permeable and a competitive inhibitor of HIF-hydroxylases, thereby enabling stabilization and heterodimerization of HIF-1α [8]. In order to apply hypoxia to desired areas within the construct, DMOG has to be delivered by a suitable vehicle. Recently, nanoporous silica nanoparticles (NPSNPs, or mesoporous silica nanoparticles, MSNs) have become a prominent tool for drug delivery, as they offer several advantages for medical applications. Their properties, such as degradability and particle size are easy to tune and their physicochemical properties, such as small pore size, large surface area and large pore volume allow the delivery of a variety of different drugs and molecules [9–11].

In the past years, fibrin, which is a polymer of the fibrinogen monomer, has become one of the most prominent biomaterial sources for the fabrication of hydrogels. It is known to be naturally angiogenic, offers a high number of cell adhesion sites and its sources can either be autologous, allogeneic, xenogeneic or recombinant [12, 13]. Moreover, fibrin gels have found several applications in tissue engineering approaches of cardiovascular tissue, bone, adipose and skin tissue [14–17].

Here, we investigated whether DMOG-loaded NPSNPs are suitable to induce hypoxic conditions in fibrin hydrogels with the aim to support endothelial tube formation by sustained VEGF secretion from ASC. For this purpose, DMOG loading capacity and release from NPSNPs were characterized. VEGF secretion profiles from ASC after treatment with solubilized and NPSNPs-delivered DMOG were compared and the latter was incorporated in tube formation assays with EC and ASC to test their effect on characteristic tube parameters.

## Materials and methods

### Cell culture

ASC were isolated from abdominal subcutaneous adipose tissue of one 31-year-old female donor (donor 5 in Ref. [18]) and one 37-year-old female donor, both scheduled for visceral surgery after ethical approval of the institutional review board (Ethikkommission der Medizinischen Hochschule Hannover) and informed consent.

### Treatment of ASC with solubilised DMOG

ASC were seeded into wells of a 96-well plate at a density of 10 000 cells/well and cultured in Dulbecco's Modified Eagle's Medium (DMEM) containing 10% Fetal Bovine Serum (FBS) (Pan Biotech, Aidenbach, Germany), 1% penicillin/streptomycin (Sigma, Munich, Germany), 1% amphotericin B (Merck, Darmstadt, Germany), 1% gentamycin (Merck, Darmstadt, Germany), 1% glutaMax™-I (Life Technologies, Darmstadt, Germany) and 10 ng/mL Fibroblast Growth Factor (FGF) (Peprotech, Hamburg, Germany) (ASC complete media) overnight at 37°C and 5% CO_2_. On the following day, cell culture medium was aspirated from wells, and cells were treated with ASC complete media containing 100 and 500 µM DMOG (Merck, Darmstadt, Germany). Groups tested were (i) single dosage of 100 and 500 µM DMOG on day 0 for three days with media change to ASC complete media on days 3 and 6, and (ii) repeated dosages of 100 and 500 µM DMOG on day 0, 3 and 6 (*n* = 3 in biological replicates). Cells receiving only ASC complete medium on day 0, 3 and 6 were used as a negative control. Medium supernatants were aspirated on day 3, day 6 and day 9 and stored at −20°C for later VEGF analysis by ELISA. At each time point, wells were filled with 110 µL DMEM without serum containing 10 µl WST-8 (Promokine, Heidelberg, Germany) and cells were tested for their viability by measuring absorption at 450 nm after 2 h.

### Incubation of ASC under hypoxic conditions

ASC were cultured as mentioned above (*n* = 9 in biological replicates). After normoxic incubation, the cells were transferred to a hypoxic chamber at 0.2% O_2_ and 37°C and incubated for 24, 48 and 72 h. Supernatants were taken off after each time point and stored at −20°C for later VEGF analysis by ELISA. As also performed with DMOG-treated cells, wells were filled with 110 µL DMEM without serum containing 10 µl WST-8 and cells were tested for their viability by measuring absorption at 450 nm after 2 h.

### Fabrication of NPSNPs, DMOG loading, and determination of loading and release efficiency

NPSNPs were fabricated according to a modified protocol by Qiao *et al*. [19], as previously published by Schmidt *et al*. [20]. Briefly, 3.16 g cetyltrimethylammonium bromide (Sigma, Munich, Germany) and 0.23 g diethanolamine (Sigma, Munich, Germany) were added to a solution of 75 ml ultrapure water and 13.4 ml absolute ethanol (Merck, Darmstadt, Germany). The solution was heated to 40°C while stirring and 8.56 ml tetraethyl orthosilicate were added after 30 min, the mixture was allowed to stir for additional 2 h. The resulting nanoparticles were then centrifuged at 18 000 g for 30 min, washed twice with water and once with ethanol, and dried overnight at 60°C. Afterwards, the organic material was removed by calcination at 550°C for 5 h with a heating rate of 1 K/min. The resulting nanoporous silica nanoparticles were monodisperse with a diameter of about 45 nm and a pore size of 3 nm. The Brunauer-Emmett-Teller (BET) surface area was determined with a value of about 950 m^2^/g by nitrogen sorption investigation as shown in Supplementary Fig. 1 and the publication cited in this respect (see Ref. [20]).

Additionally, an amino modification of the silica surface was performed by post-grafting. For this purpose, 200 mg of NPSNPs were dispersed in 8 ml toluene, and 40 µL 3-aminopropyl triethoxysilane (Sigma, Munich, Germany) were added. The solution was stirred for 2 h at 80°C and subsequently for 22 h at room temperature (RT). The modified NPSNPs were then collected via centrifugation, washed thrice with ethanol and then dried at 60°C. The success of the amino modification was proven by pH-dependent zeta potential measurement as presented elsewhere [20]. The amino-modified silica nanoparticles have a strong positive zeta potential (>20 mV) at pH values below approximately 6.5 and an isoelectric point of 7.4. This positive charge results from the protonation of amino groups on the surface. A further hint for a successful amino modification of the NPSNPs can be found in the results of the nitrogen sorption investigations. The BET surface area calculated from the isotherms decreases from a value of about 950 m^2^/g for the unmodified NPSNPs to about 330 m^2^/g for the amino-modified NPSNPs (concerning pore volume from 1.0 cm^3^/g to 0.4 cm^3^/g), which shows that a high nanoporosity is still present. This reduction of surface area and pore volume while the average pore size of 3 nm for both materials remains unaffected is caused by partial pore blocking, which might result either from a slight dissolution/reprecipitation of the nanoparticles during the modification treatment or from the deposition of organosilane residues on the particle surface, which can be formed by self-condensation of the silanization reagent in solution.

The loading efficiency of DMOG on amino-modified NPSNPs was determined via thermogravimetry using the following formula:

loading efficiency%=loaded DMOG amount mgoffered amount mg×100

Here, 5 mg NPSNPs were loaded with DMOG at a concentration of 5 mg/mL DMOG (offered amount) in a volume of 1 ml. NPSNPs were incubated with reconstituted DMOG in ddH_2_O at 4°C, shaken at 1000 rpm for 4 h and subsequently centrifuged at 4°C at 10 000 rpm for 10 min, and washed once more with ddH_2_O. Afterwards, thermogravimetric analysis (TGA) was measured with a Netzsch STA 429 CD analyser, where samples were heated up to 1000°C under air atmosphere at a heating rate of 5 K/min. The Proteus Thermal Analysis 4.3.1 program from the instrument manufacturer was used for subsequent evaluation of data.

To test the release efficiency of NPSNPs, they were loaded with DMOG as described above. After loading, NPSNPs were incubated in 1 ml ddH_2_O and incubated at 37°C. Every day for 6 days, NPSNPs were centrifuged and the supernatant was taken off. After 1 ml fresh ddH_2_O was added, NPSNPs were shortly vortexed and again incubated at 37°C. Supernatants were taken off and stored at −20°C and later analysed by measuring the UV/Vis absorption at 430 nm.

### Treatment of ASC with DMOG-loaded NPSNPs

Sterile NPSNPs were loaded with DMOG as described before and resuspended in ASC complete medium after centrifugation at 4°C at 10 000 rpm for 10 min. 10 000 ASC/well were cultured as before and treated with 0.276 mg/mL and 1.38 mg/mL freshly DMOG-loaded NPSNPs (*n* = 9 in biological replicates) to yield a DMOG release of 100 µM and 500 µM per well. Treatment strategy was the same as with solubilized DMOG: (i) single dosage of 0.276 mg/mL and 1.38 mg/mL DMOG-loaded NPSNPs, respectively, on day 0 for three days, with media being changed to ASC complete media on day 3 and 6, and (ii) repeated dosages of 0.276 mg/mL and 1.38 mg/mL DMOG-loaded NPSNPs on day 0, 3 and 6. Cells receiving no NPSNPs at the respective time point(s) served as negative control. As before, medium supernatants were aspirated on day 3, day 6 and day 9. For long-term treatment with DMOG-loaded NPSNPs, cells were further treated for 15 additional days with supernatants being aspirated every third day. All supernatants were stored at −20°C for later VEGF analysis by ELISA and cell viability was assessed by WST-8 assay on the respective time points.

### VEGF ELISA

Produced and released amount of VEGF from ASC were determined by using an 2,2′-azino-bis(3-ethylbenzothiazoline-6-sulfonic acid (ABTS) ELISA Kit (Peprotech, Hamburg, Germany), following the protocol supplied by the company. Briefly, plates were coated with capture antibody overnight, the standard and samples (diluted 1:10) were added the next day after blocking and incubated for 2 h. After washing, plates were incubated with detection antibody for 2 h. The avidin-HRP conjugate was added to wells afterwards and after 30 min of incubation, the ABTS liquid substrate was added. Plates were incubated for 40–50 min in the dark at RT and absorbance was subsequently measured at a wavelength of 406 nm with a reference of 650 nm.

### Tube formation assay with DMOG-loaded NPSNPs

To test tube formation ability of EC under the influence of DMOG and NPSNPs, a tube formation assay was performed. Groups tested were: (i) EC-ASC co-culture without DMOG and NPSNPs (Control), (ii) EC-ASC co-culture supplied with 100 µM or 50 µM DMOG on NPSNPs and (iii) EC-ASC co-culture with unloaded NPSNPs (*n* = 12 in biological replicates). For each well, 50 µL of fibrinogen were mixed with 1 µL of a 2.75 U/mL thrombin solution (CSL Behring, Marburg, Germany) to give a final fibrin concentration of 5 mg/mL, pipetted into wells of a 96-well plate and allowed to polymerize for 30 min. Following polymerization, gels were seeded with red-fluorescent protein-expressing human umbilical vein endothelial cells (RFP-HUVEC) (Pelo Biotech, Planegg/Martinsried, Germany) and ASC, with a cell number of 4000 EC/well and 2000 ASC/well. Wells were supplied with 200 µL M199 feeding media containing FGF (40 ng/mL), VEGF (40 ng/mL) and aprotinin (100 U/mL; all from Sigma) and incubated at 37°C with 5% CO_2_ for 7 h to allow cell attachment. In the meantime, NPSNPs were loaded with DMOG as mentioned above. After loading, NPSNPs were centrifuged at 10 000 rpm at 4°C and resuspended in Phosphate Buffered Saline (PBS). Feeding medium was aspirated and the second gel layer was added to the wells, either holding no, or 13.9 µg (for 100 µM DMOG) or 6.95 µg (for 50 µM DMOG) unloaded or DMOG-loaded NPSNPs/well. Gels were allowed to polymerize for 20 min and subsequently overlaid again with feeding medium. Medium was exchanged every 2 days for an assay duration of 7 days. Tube formation was characterized by AngioTool Software 0.6a [21] after 4 and 7 days analysing tube length, tube area and number of junctions in each group.

### Live/dead staining

ASC were cultured and treated as described in the section ‘Treatment of ASC with solubilized DMOG’. At each time point, supernatants were taken off, cells were washed twice with PBS and subsequently stained with 1 µM Ethidium Homodimer-1 and 0.5 µM Calcein AM from a live/dead staining kit (Invitrogen, Darmstadt, Germany) (*n* = 9 in biological replicates). After an incubation of 45 min, staining solution was aspirated, wells were filled with PBS and images of stained cells were obtained by fluorescence microscopy.

### Statistics

Statistical analyses were performed using Graphpad Prism 6.04 (Graphpad Software, San Diego, California). Normal distribution of the data was tested using the d’Agostino & Pearson omnibus normality test. Results are presented as mean ± SEM unless otherwise specified in figure legends. Multiple comparisons between groups were performed by two-way ANOVA followed by Bonferroni’s post-test. Differences were considered significant at *P* < 0.05. Significance levels were given as follows: ^§^: marginally significant, *P* ≥ 0.05; *: *P* < 0.05; **: *P* < 0.01, ***: *P* < 0.001 and ^#^: *P* < 0.0001.

## Results

### Treatment of ASC with solubilized DMOG

**Figure 1.**
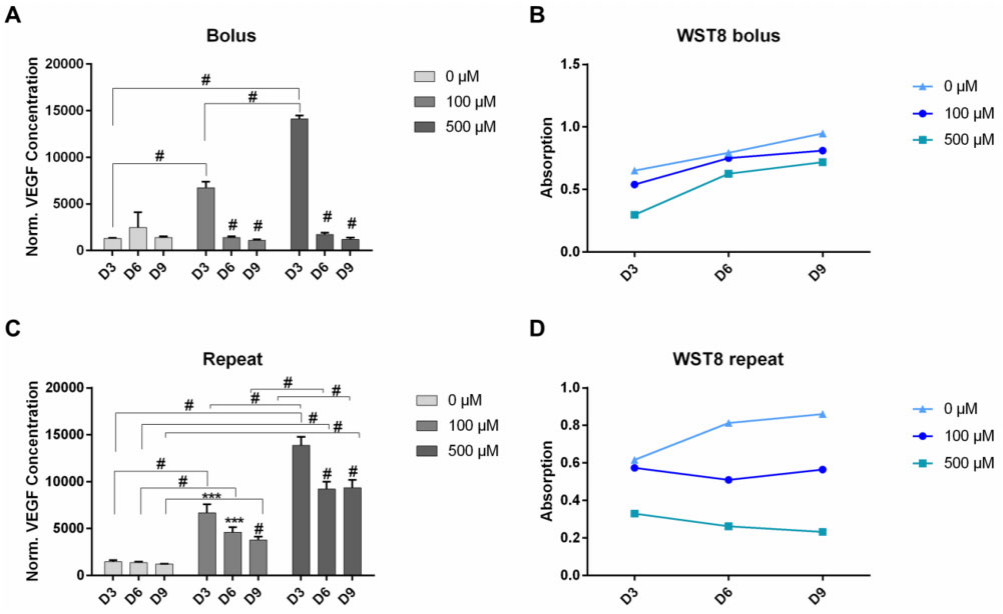
VEGF production of ASC after treatment with solubilized DMOG up to 9 days. Cells were either treated with a single dose (**A**) or with repeated doses (**C**) of DMOG in the indicated concentrations. After treatment cell viability was assessed by WST-8 assay (**B and D**). Shown are mean ± SD with biological replicates of *n* = 3 for each group ***: *P* < 0.001 and #: *P* < 0.0001 by two-Way ANOVA and Tukey’s post-test

To increase VEGF production of ASC, cells were treated with solubilized DMOG of different concentrations either in single or repeated dosages. All cells treated with DMOG showed increased levels of VEGF production (Fig. 1A). For cells treated with a single dose of 100 µM DMOG, VEGF production slightly increased until day 3, significantly decreased however on day 6 and day 9 (*P* < 0.0001). VEGF production of cells treated with a single dose of 500 µM DMOG significantly increased until day 3 (*P* < 0.0001), with VEGF amounts being 2.1-fold higher than in cells treated with 100 µM. However, VEGF secretion also with this concentration significantly decreased on days 6 and 9. The viability of cells treated with single doses of 100 µM and 500 µM DMOG did not decrease noticeably over the assay duration, however, cells treated with 500 µM showed the lowest viability (0.76-fold lower on day 9 compared to the control) (Fig. 1B).

Due to the finding that a single treatment of cells with DMOG is not enough to sustain the release of VEGF in ASC, cells were treated repeatedly with DMOG at different concentrations. In this case, a slightly sustained VEGF production could be achieved, with levels being 3-fold higher (*P* < 0.0001) in cells treated with repeated doses of 100 µM DMOG and 7.6-fold higher (*P* < 0.0001) in cells treated with repeated doses of 500 µM DMOG on day 9. VEGF production in cells treated repeatedly with 500 µM DMOG was significantly higher compared to cells treated with repeated doses of 100 µM DMOG (*P* < 0.0001), with VEGF amounts being 2- to 2.5-fold higher over the duration of 9 days of the assay (Fig. 1C). Although VEGF secretion was significantly decreased in all groups treated with repeated doses of DMOG from day 3–6, it could still be maintained at higher levels, especially in the group treated with 500 µM DMOG, as compared to groups treated with only a single dose of DMOG. Moreover, VEGF amounts did not significantly decrease between day 6 and day 9.

While repeated treatment of 500 µM DMOG yielded the highest secretion amounts of VEGF, it could also be observed that cell viability in this group steadily decreased over the assay duration of 9 days (Fig. 1D). Compared to this, for cells treated with a repeated dosage of 100 µM cell viability could be better sustained until day 9. Thus, although, this dosage yielded a slightly lower increase in VEGF production, we considered a 100 µM dosage to be more suitable considering the goal of long-term VEGF release.

To test, whether physically induced hypoxia would result in similarly elevated VEGF levels in ASC, cells were incubated for 24, 48 and 72 h at 0.2% O_2_ (Supplementary Fig. 2A). Results after ELISA analysis of supernatants showed that VEGF production of cells incubated at 0.2% O_2_ increases gradually over time, with values after 24 h of hypoxic incubation already being 2.2-fold higher than values of the control (*P* < 0.01). VEGF levels after 72 h of hypoxic incubation were 2.4-fold (*P* < 0.01) and 1.3-fold (*P* < 0.0001) higher than values after 24 and 48 h hypoxic incubation, respectively. Moreover, VEGF levels of cells treated with hypoxia for 72 h are indeed comparable to VEGF levels obtained in cells treated with 100 µM DMOG for 3 days. Cell viability of cells under hypoxia for 24 h was higher than viability of cells of the control and even slightly increased between 24 h and day 48 h. However, viability of control cells kept on increasing over time until 72 h, while viability of hypoxic ASC slightly decreased between 48 h and 72 h (Supplementary Fig. 2B).

A live/dead staining of DMOG-treated ASC was also performed. The staining verified WST-8 measurements, showing that the number of dead ASC was the highest on day 3 in groups treated with DMOG. The number of dead cells was only lowered when cells did not receive another DMOG dose after day 3 and day 9 (Supplementary Fig. 3). For cells treated repeatedly with 100 µM DMOG, live/dead staining showed a relatively constant number of dead cells, which was significantly higher on day 6 (*P* < 0.01) and on day 9 (*P* < 0.001) (Supplementary Fig. 3B), which is also in correlation to WST-8 data from this group. However, staining results from cells treated repeatedly with 500 µM DMOG appear rather fluctuating, with numbers of dead cells being significantly higher (*P* < 0.05) on day 9 than on day 6, while WST-8 values on day 9 are lower than on day 6.

### Loading and release efficiency of DMOG on NPSNPs

**Figure 2.**
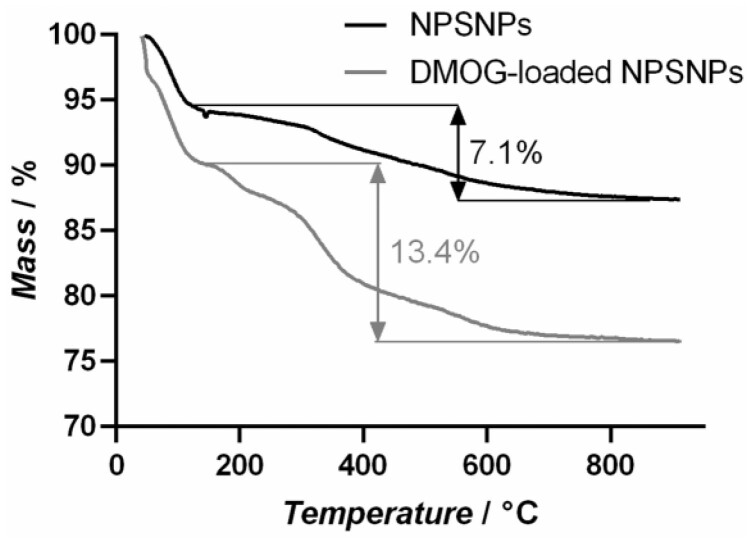
Thermogravimetric measurement assessing loading efficiency of NPSNPs. 5 mg unloaded and 5 mg DMOG-loaded (5 mg/mL) NPSNPs were heated up to 1000°C, leading to combustion of organic residues and condensation of the silica network for unloaded NPSNPs. DMOG-loaded NPSNPs exhibit an additional mass loss due to combustion of DMOG. Comparing the curves, a loading efficiency of 1.3% can be calculated

The loading efficiency was determined by thermogravimetric measurements (Fig. 2). For this purpose, DMOG-loaded and non-loaded NPSNPs were heated up to 1000°C and the mass loss was detected. After losing weight due to adsorbed water, the non-loaded NPSNPs show a mass loss of 7.1% due to combustion of the organic aminopropyl residues on the surface introduced by modification and further condensation of the silica network. Within this condensation reaction, silanol (–Si–OH) groups are forming siloxane (–Si–O–Si–) bridges. The DMOG-loaded NPSNPs exhibit a mass loss of 13.4% in this temperature range. Here, the mass loss is evoked by simultaneous effects, which are the combustion of organic residues further silica condensation reaction, and the combustion of the DMOG on the outer and inner surface of the NPSNPs. Assuming that the mass loss due to combustion of organic residues and further condensation of the silica network is equal in DMOG-loaded and non-loaded NPSNPs, 6.3% of the mass loss can be ascribed to the DMOG combustion, which leads to a value of 63 µg DMOG loaded on one milligram of NPSNPs and loading efficiency of 1.3%.

**Figure 3.**
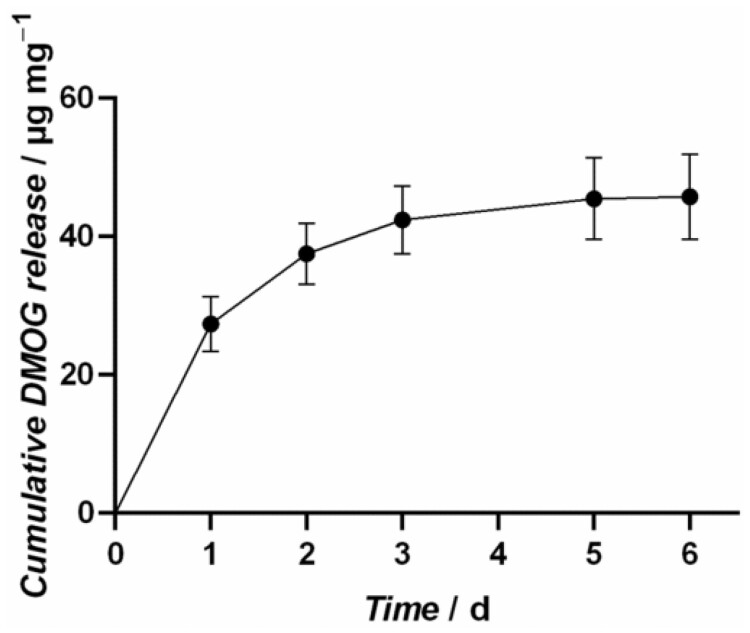
Release profile of DMOG-loaded NPSNPs. 5 mg particles were loaded with 5 mg/mL DMOG and incubated in H_2_O for 6 days, showing that 43% of the loaded DMOG amount was released within the first day. In total, 73% of the loaded DMOG amount are released from the particles after 6 days

The amounts of DMOG released from the NPSNPs were investigated in a release experiment in water at 37°C over 6 days. The DMOG-loaded NPSNPs are showing a continuous release profile as illustrated in Fig. 3. It can be stated that the largest fraction of 43% of the loaded DMOG amount, corresponding to 27 ± 4 µg DMOG per mg NPSNPs, are released within the first day. In the following, remarkable portions of 10 ± 0.4 µg DMOG per mg NPSNPs and 5 ± 0.5 µg DMOG per mg NPSNPs are released in time interval two (24 h) and three, respectively. Overall, about 73%, 46 ± 6 µg DMOG per mg NPSNPs, of the loaded DMOG amount are released after 6 days. As the detected amounts of DMOG after 6 days were under the quantification limit, the release experiment could not be performed for a longer time period.

### Treatment of ASC with DMOG-loaded NPSNPs

After testing the effect of solubilized DMOG on cells, also DMOG-loaded NPSNPs were tested on ASC to evaluate, whether the controlled release of DMOG from the particles could sustain VEGF production at higher levels over the whole assay duration. Again, cells were treated either with single or repeated dosages of DMOG-loaded NPSNPs to a final concentration of 100 µM or 500 µM DMOG.

**Figure 4.**
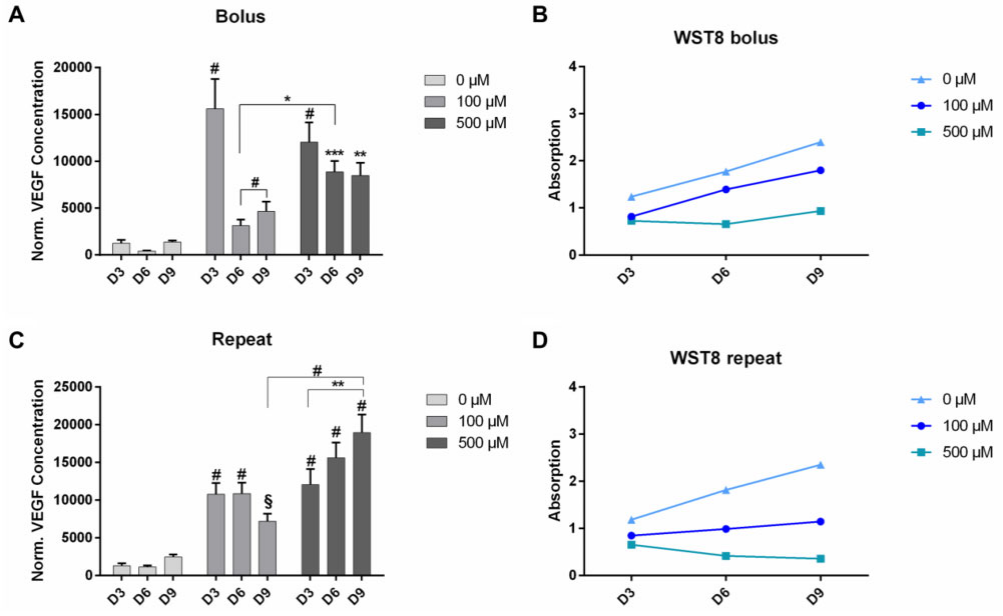
VEGF production of ASC after treatment up to 9 days with DMOG-loaded NPSNPs. Cells were either treated with a single dose (**A**) or with repeated doses (**C**) of dimethyloxalylglycine (DMOG)-loaded NPSNPs in the indicated concentrations. After treatment, cell viability was assessed by WST-8 assay (**B and D**). Shown are mean ± SEM with biological replicates of *n* = 9 for each group. §: *P* < 0.063, *: *P* < 0.05; **: *P* < 0.01; ***: *P* < 0.001 and #: *P* < 0.0001 by two-way ANOVA and Tukey’s post-test

On day 3, a significant increase in VEGF production could be seen in cells treated with a single dosage of 100 µM DMOG (*P* < 0.001) with VEGF levels being 12.7-fold higher than in cells of the control and 1.3-fold higher than in cells treated with of 500 µM (Fig. 4A). However, VEGF levels in this group decreased until day 6 with a slight recovery until day 9, while interestingly the cell viability kept increasing at the same time. VEGF production in cells treated with a single dosage of 500 µM DMOG was still significantly higher than in cells of the control (9.8-fold higher, *P* < 0.01). What is striking in this group is, however, that VEGF levels did not decrease as drastically on day 6 as they did in cells treated with 100 µM of DMOG but instead could also be rather sustained until day 9, while at the same time cell viability was the lowest of all groups and did not increase remarkably until day 9 of the assay (Fig. 4B).

When cells were treated with repeated dosages of DMOG-loaded NPSNPs again a significant increase in VEGF production could be observed in both treated groups on day 3 (*P* < 0.001), with VEGF levels being approximately 8.7-fold higher when treated with 100 µM DMOG and 9.7-fold higher when treated with 500 µM DMOG compared to levels of the control group on day 3. In cells treated with repeated dosages of 100 µM DMOG, it was observed that VEGF production could be sustained at significantly higher levels compared to the control until day 9 (marginally significant with *P* = 0.063) with cell viability staying constant throughout the whole assay duration (Fig. 4C). Interestingly, VEGF production in cells treated repeatedly with 500 µM even delivered increasing amounts of VEGF until day 9, with levels on day 9 being 7.7-fold higher (*P* < 0.0001) compared to control and 1.6-fold higher (*P* < 0.01) than on day 3 and significantly higher than levels on day 9 of cells treated with 100 µM (*P* < 0.0001). However, it could be recognized again that cell viability in this group was the lowest and decreased even more until day 9 (Fig. 4D).

Considering these results, another experiment was performed, testing the effect of long-term treatment of cells with repeated dosages of 500 µM DMOG-loaded NPSNPs (Supplementary Fig. 4) and whether VEGF production could be sustained even for a longer time. Here, although VEGF levels shortly decreased until day 6, a clear trend of increasing VEGF levels until day 12 could be observed. Whereas VEGF production in cells of the control kept increasing until day 18 of the assay, VEGF levels of cells treated with DMOG were significantly decreased on day 15 and even lower on day 18 (*P* < 0.001). Cell viability of cells of the control increased throughout the whole assay duration, but could only be depicted as a plateau after day 9, since absorbance values were out of detection range. Viability for treated cells was again low and further decreased between day 15 and day 18.

### Tube formation assay with DMOG-loaded NPSNPs

**Figure 5.**
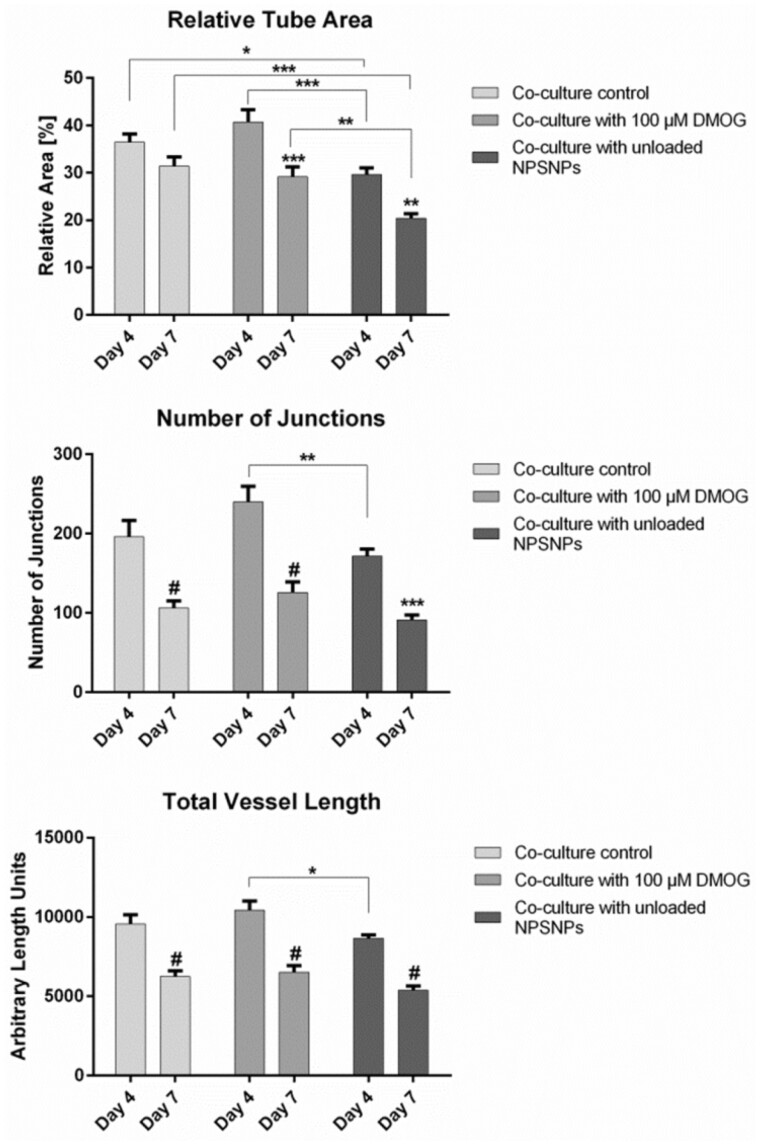
AngioTool analysis of tube parameters of the endothelial vascular network in tube formation assay supplied with 100 µM DMOG on NPSNPs. Parameters analysed were relative tube area (**A**), number of junctions (**B**) and total vessel length (**C**). Values were compared between day 4 and day 7 of the culture. Shown are means ± SEM of four biological replicates run in triplicates. *: *P* < 0.05, **: *P* < 0.01, ***: *P* < 0.001 and #: *P* < 0.0001 by two-way ANOVA and Tukey’s post-test

To test whether the application of unloaded or DMOG-loaded NPSNPs would interfere with the tube formation capabilities of EC, a tube formation assay in fibrin hydrogel was performed. As shown in Fig. 5, tube analysis by AngioTool revealed a decrease in all tube parameters, which are tube length, tube area, and number of junctions, in the control group between day 4 and day 7. This effect was even more distinct in wells receiving 13.9 µg of unloaded NPSNPs, which can also be seen in Supplementary Fig. 5. Same results were obtained in wells receiving 100 µM DMOG on NPSNPs (Supplementary Fig. 5), where a decline in all parameters evaluated was seen. The most distinct effect was the decrease in number of junctions between day 4 and day 7 (1.9-fold) (Fig. 5).

**Figure 6.**
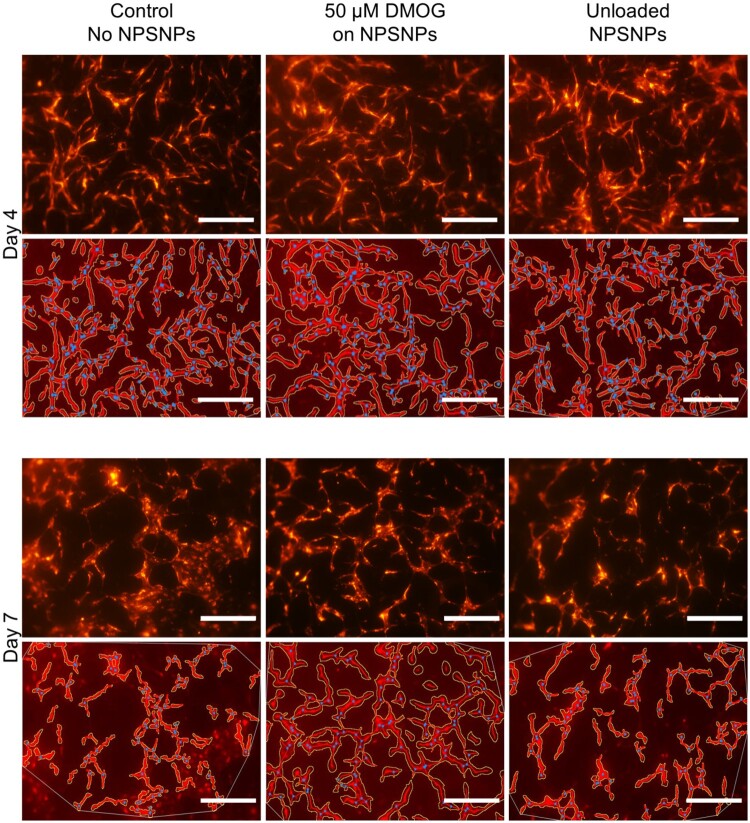
Endothelial tube formation of RFP-HUVEC in fibrin hydrogels of 5 mg/mL fibrinogen concentration treated with DMOG-loaded NPSNPs. Wells with EC-ASC co-culture were supplied with 50 µM DMOG loaded on NPSNPs and unloaded NPSNPs after day 4 and day 7 of the assay, shown in the upper rows of each time point respectively. Parameters of the vascular network, that are relative tube area, number of junction, and tube length, were analysed by AngioTool, images after analysis are shown in lower rows for each time point, respectively. Vascular networks were masked and outlined and points of junction marked in blue dots by the software. Scalebar: 500 µm

Considering these results, the DMOG concentration was decreased to 50 µM (Fig. 6).

**Figure 7.**
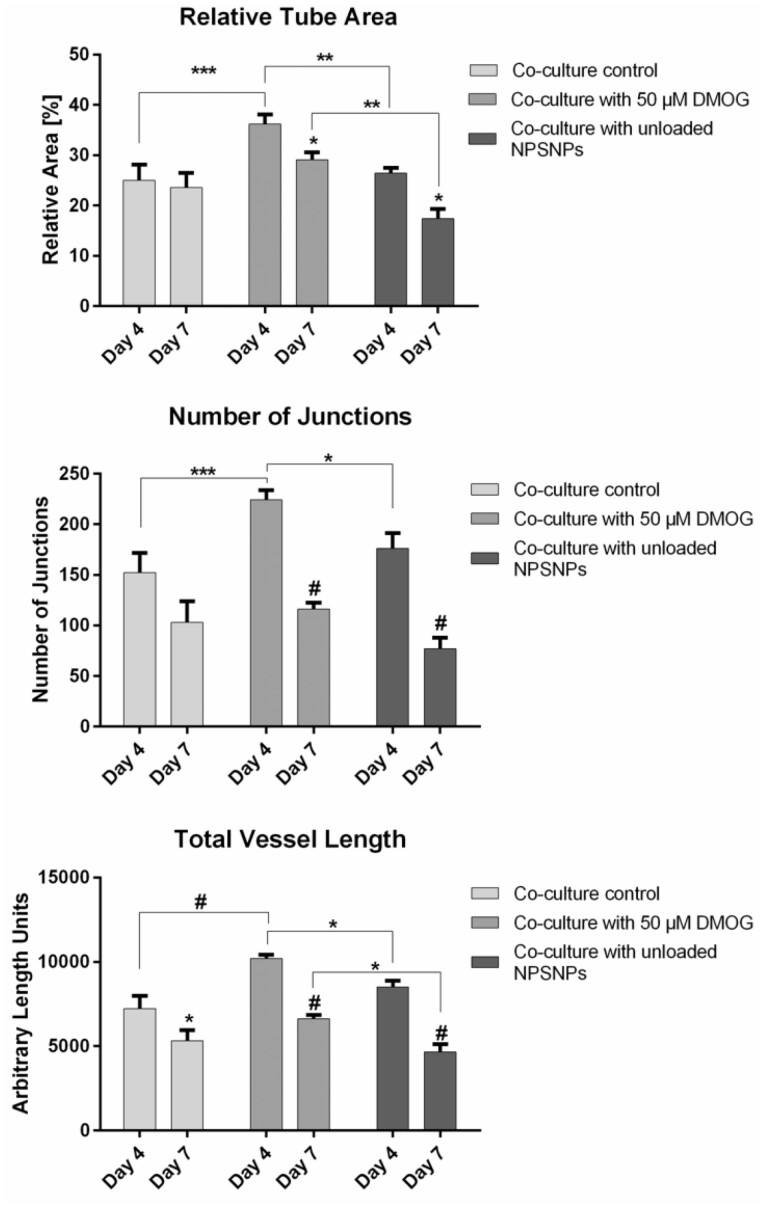
AngioTool analysis of tube parameters of endothelial vascular network in tube formation assay supplied with 50 µM DMOG on NPSNPs. Parameters analysed were relative tube area (**A**), number of junctions (**B**) and total vessel length (**C**). Values were compared between day 4 and day 7 of the culture. Shown are means ± SEM of four biological replicates run in triplicates. *: *P* < 0.05, **: *P* < 0.01, ***: *P* < 0.001 and #: *P* < 0.0001 by two-way ANOVA and Tukey’s post-test

Under this condition, AngioTool analysis of tubes on day 4 showed a significant increase in all parameters tested in groups treated with either DMOG-loaded or unloaded NPSNPs compared to wells of the control (Fig. 7). In wells treated with 50 µM DMOG, a 1.5-fold higher average tube area, 1.5-fold higher number of junctions, and 1.4-fold higher total vessel length was achieved compared to the untreated control, which could also be visualized in Fig. 6. In wells treated with unloaded NPSNPs, average tube area was 1.1-fold, number of junctions 1.2-fold, and total vessel length 1.2-fold higher than in wells of the control (Fig. 7). However, again a significant decrease in all parameters and all groups was observed from day 4 to day 7 of the assay, with values of groups treated with DMOG-loaded and unloaded NPSNPs being on approximately the same levels as values of the control group. We here want to point out that although absolute values of tube parameters of cells treated with 50 µM DMOG are comparable to absolute values of cells treated with 100 µM DMOG, we thought the results of treatment with 50 µM DMOG to be more reliable and sound, since significant differences were achieved in all parameters tested. This could not be shown in the treatment with 100 µM DMOG.

Consequently, DMOG released from NPSNPs significantly improved EC tube formation in short timeframe, resulting in quicker formation of more complex early tube networks. This effect was transient and did not delay tube degradation compared to the controls.

## Discussion

One of the major goals of tissue engineering is the generation of a fully cellularized tissue construct that can integrate and become metabolically and functionally active *in vivo*. One of the biggest obstacles being faced here is the inadequate supply of oxygen and nutrients to the cells within the tissue construct, which leads to failing tissue integration [22]. Therefore, tissue-engineered constructs need a pre-formed vascular network integrated, in order to enable perfusion and nutrient delivery also to deep layers of the construct. Capillary tube formation is dependent on several factors, including VEGF. Since a repeated delivery of fresh VEGF to the tissue construct in long-term *in vitro* or even *in vivo* applications is rather impractical, there is a need for sustained VEGF supply and delivery. Therefore, the aim of this study was to determine whether VEGF production of ASCs can be increased when treated with DMOG and moreover, whether it can be prolonged and sustained when DMOG is controllably delivered via NPSNPs over a time period of 9 days. Furthermore, our results show first steps towards the generation of a pre-capillarized tissue construct holding a co-culture of RFP-HUVEC and ASC in the presence of DMOG-loaded NPSNPs.

The major findings of this study are (i) ASC show sustained release of VEGF for up to 12 days without comprising cell viability when treated repeatedly with DMOG-loaded NPSNPs, and (ii) DMOG-loaded NPSNPs supported tube formation of EC when co-cultured with ASC.

### Effect of solubilized DMOG on ASC

Blood vessels in the adult body form a highly branched network following a structural hierarchy. Overall, every blood vessel is formed by EC, which are supported by mural cells, which can either be pericytes for small vessels like capillaries, or smooth muscle cells for bigger vessels like arteries [23]. Mural cells promote stability, control permeability and secrete VEGF as a survival cue for the inner-lying endothelium [23, 24]. Verseijden *et al*. [25] have shown that mesenchymal stem cells, bone marrow, as well as adipose tissue-derived, can develop into mural cells when co-cultured with EC. ASC are of particular interest in these applications, as large cell numbers can be obtained rather easily, but more importantly, they produce pro-angiogenic factors (i.e. VEGF) and can develop pericyte characteristics [24–26]. ASC are known to reside in hypoxic niches with O_2_ levels under 4% and it is therefore assumed that culturing ASC under low oxygen conditions might support their proliferation, growth factor secretion and viability [27, 28]. In this study, chemically induced hypoxia was chosen over physically induced as a later goal of the project is to direct hypoxic environments within specific areas of the hydrogel construct. Since this is hard to achieve using hypoxic chambers, as hypoxia would be ubiquitous, we used the opportunity of loading DMOG onto NPSNPs and thereby being able to introduce hypoxia in selected zones. DMOG treatment stabilizes HIF-1α, triggering the transcription of various hypoxia-sensitive genes among which VEGF is the most prominent. As shown in Fig. 1, VEGF production of ASC could indeed be significantly increased in a short-term manner, when exposing the cells to 100 µM and 500 µM of solubilized DMOG in single-dose treatment. However, one dose of DMOG in either concentration did not stabilize the VEGF secretion of cells over a longer time period, presumably because DMOG was hydrolysed too quickly in the medium to achieve long-term effects. Hydrolysis of DMOG is known to result in NOG, which is not able to penetrate the cell membrane due to its high hydrophilicity and could therefore not act on HIF-1α stabilization if present in the medium. After DMOG entered the cell, however, it is de-esterified to NOG, which can not only act on PHDs to stabilize HIFs, but also on α-KG-dependent processes, which is known to play an important role in cell metabolism [8]. Although the manipulation of these processes might lead to decreased cell viability, the specific effects of DMOG on α-KG-dependent processes still need to be further investigated [8]. Interestingly, it has also been shown that a rapid and spontaneous mono-de-esterification of DMOG occurs in media, which leads to the production of methyloxalylglycine (MOG) [29]. MOG can easily be transported into cells by using monocarboxylate transporter 2 (MCT2), where it is also further de-esterified and leads to the accumulation of NOG in the millimolar range, which is far higher than what is needed for the inhibition of PHDs [29]. However, this study was performed in cancer cells and it needs to be investigated, whether similar effects could be observed in mesenchymal stem cells.

Another point of interest was to investigate, whether physically induced hypoxia would result in similarly elevated VEGF levels as in DMOG-treated cells. After incubating ASC in a hypoxic chamber at 0.2% O_2_ for up to 72 h, we could show that VEGF levels of cells incubated at hypoxic conditions for 72 h indeed showed comparable VEGF levels as cells treated with 100 µM DMOG. A longer incubation time at these hypoxic conditions could not be performed, as cells would not have survived this. Nevertheless, we want to conclude that physically induced hypoxia leads to VEGF levels that can be compared to results of DMOG treatment. However, a point that still needs to be evaluated is whether elevation in VEGF secretion upon DMOG treatment was indeed induced by HIF stabilization, or possibly by other, HIF-independent pathways.

In our experiments, we observed a decrease in cell viability, which could only be recovered when no DMOG was added after day 3 of the assay. Both effects, VEGF release and decreased viability, were more pronounced when exposing cells to repeated doses of 100 µM and 500 µM solubilized DMOG (Fig. 1). Although cell viability was low, VEGF production could still be slightly maintained. We want to point out that a concentration of 100 µM DMOG was sufficient to induce increased and maintained VEGF secretion, while at the same time protecting cells from a significant loss of viability. We here want to mention that it has to be taken into consideration, how cell viability is to be evaluated when treating cells with DMOG. As also reviewed by Nelson *et al*. [8], DMOG is known to interfere with glutamate dehydrogenase and isocitrate dehydrogenase, thereby possibly leading to disturbances in the cell cycle and NAD/NADH levels. As our chosen WST-8 cell viability kit tests for dehydrogenase activity, we performed additional live/dead staining, in order to confirm validity of the before measured values (Supplementary Fig. 3). The staining indeed verified WST-8 measurements for cells treated with a single dose of DMOG and for cells treated with repeated doses of 100 µM, while the number of dead cells of cells treated repeatedly with 500 µM DMOG obtained after live/dead staining did not fully support WST-8 values. While we still believe our results to be properly represented by choosing WST-8 measurement, this staining result should underline the importance of choosing the right test to investigate cell viability.

Interestingly, our results differ in some aspects from findings of other studies investigating DMOG treatment on ASC. While Chen *et al*. [30] have also found 100 µM DMOG to be the optimal concentration for short-term ASC pre-treatment, they have also reported higher survival and reduced cell death rates. Ding *et al*. [31] have found an increase in VEGF release from ASC for at least 28 days, as well as a slight decrease in proliferation in a dose-dependent manner. However, they did not observe differences in cell viability after treatment with 200 µM, 500 µM and 1000 µM DMOG. DMOG and its effects have also been investigated in other studies utilizing a variety of cell types, many of them presenting rather controversial results [32–34]. This leads to the conclusion that the effect of DMOG possibly depends strongly on the cell type and culture conditions chosen. It should be noted, however, that to the best of our knowledge, only very few studies have utilized ASC when determining the effects of DMOG.

### Effect of DMOG-loaded NPSNPs on ASC

Considering our first findings, we assumed that the experimental setup with solubilized DMOG displays a strong challenge for cells and that the hypoxic environment needed to be established in a more controlled manner. The use of NPSNPs offers a great opportunity for the delivery of soluble factors and molecules. It was shown that the loading capacity was 63 µg DMOG/mg NPSNPs and that up to 73% of DMOG were delivered in the time course of 6 days. Thus, DMOG-loaded NPSNPs appeared to be an ideal candidate to expose ASC to sustained chemical hypoxia in a controlled manner. NPSNPs concentrations chosen exposed the cells to 100–500 µM DMOG, delivered to ASC either as single or repeated doses in a short-term experiment of 9 days (Fig. 4), as well as in a long-term experiment over 18 days (Supplementary Fig. 4). As with soluble DMOG, also with DMOG-loaded NPSNPs the VEGF production was markedly increased, indicating that a sufficient amount of the delivered DMOG was biologically active and not hydrolysed to NOG during the release and delivery process from NPSNPs. Differently to solubilized DMOG on ASC, VEGF amounts increased until day 3 when only a single dosage of DMOG on NPSNPs was delivered to the cells while cell viability did not decrease. In contrast, it rather increased over the remaining assay duration between day 3 and day 9, which could indicate DMOG’s capability to enhance cell survival, which has also been seen in other studies [30–32]. However, it should be noted that these effects depend strongly on the cell type and culture conditions chosen. With repeated doses of DMOG from NPSNPs, moreover, it was seen that VEGF production of ASC was either maintained or even continually increased in the case of 500 µM DMOG, while only a slight decrease in cell viability was observed over the assay duration (Fig. 4). This was maintained until day 12 of the culture that seemed to display the utmost time line for DMOG-driven VEGF production (Supplementary Fig. 4) since beyond this time point cells displayed a marked loss in viability. The long-term exposure to DMOG-loaded NPSNPs on the one hand could have induced above-mentioned cytotoxic DMOG effects on cells. Too high concentrations of DMOG resulting in too high intracellular NOG concentrations and are also known to downregulate cellular respiration, which then leads to cell death [35]. On the other hand, NPSNPs themselves could have posed additional stress to the cells by toxic effects related to the surface charge of the particles or of released silica species. Although NPSNPs are generally considered as safe *in vitro* as well as *in vivo*, literature research on the cytotoxicity of NPSNPs delivers variable findings. Some studies showed that cytotoxicity of NPSNPs increased with increasing particle concentration and independently from size, treated cell type and surfactant used in the synthesis [36], while others stated that cytotoxicity increased with decreasing size of particles [37–39]. Considering these findings, NPSNPs used in this study with an average diameter of 40–50 nm should show low cytotoxicity, which is also supported by former studies using these particles [40, 41]. All these controversial findings lead to the suggestion that cytotoxicity of NPSNPs has indeed to be assessed individually for each experimental setup, considering formulations and modifications of NPSNPs, the cell types used in the study and their metabolic activity; even sterilization techniques of NPSNPs could be determining factors [42, 43].

For future studies aimed at delivering controlled hypoxic conditions to ASC, further tuning of the properties of NPSNPs should be considered, in order to prolong the controlled release and delivery of DMOG even further, so that repeated doses can be avoided. This would also be beneficial when DMOG-loaded NPSNPs are to be introduced into a cellularized hydrogel to help in the pre-vascularization of the construct, where no fresh doses of NPSNPs can be introduced after polymerization of the gel. Moreover, it has to be considered that the use of DMOG-loaded nanoparticles in cellularized hydrogels could potentially create a spatially varying hypoxia stimulus, being higher in their close proximity, as it might take longer for DMOG to evenly diffuse through the whole gel construct. If so, it has to be investigated whether these locally higher hypoxic conditions are potentially toxic to the cells, and whether the DMOG concentrations delivered to the hydrogel on NPSNPs should be considered to be lowered, due to the retention of DMOG in the gel. On the other hand, DMOG is a very small molecule as compared to many other drug molecules and should diffuse quite rapidly.

### Effect of DMOG-loaded NPSNPs on tube formation of EC

Possible retention of DMOG in a fibrin hydrogel can also be suggested from our findings when we investigated the tube formation capabilities of EC co-cultured with ASC, when DMOG-loaded NPSNPs were supplied to the system in the upper gel layer of a sandwich tube formation assay. DMOG concentrations applied in this assay were 100 µM and 50 µM. The concentration of 100 µM was chosen first, as it was seen in previous experiments that cell viability was better maintained in ASC with this concentration while upregulating VEGF secretion at the same time. To investigate the effect of DMOG on tube formation capabilities of EC, several parameters, which are generally used to describe properties of capillary network formation, were analysed. These parameters were relative tube area, describing the percentage of the well-being covered by the vascular network, number of junctions to assess the level of interconnectivity of the newly formed vasculature, and total vessel length, showing whether individual EC are simply elongated, or joining to form vascular structures. When cells were supplied with 100 µM DMOG, no significant differences in either of these categories could be observed compared to the control group (Fig. 5). More importantly, however, is to point out that significant decreases over the assay duration in all tube parameters evaluated were observed also in wells treated with 100 µM DMOG, as well as in all other groups tested, which gives room for the interpretation that DMOG at this concentration could not upregulate VEGF expression in ASC to that extent as to support endothelial tube formation for a longer time period. Another hypothesis might be that the higher VEGF levels in the tube formation assay resulting from DMOG treatment are not necessarily enough as a single factor for the sustained tube formation of EC. It is widely known that although VEGF is a potent factor in the process of vessel sprouting, it cannot induce vessel maturation alone, as was also shown by Hellström *et al*. [44–46]. Finally, there is also the possibility that 100 µM of DMOG in a tube assay might have cytotoxic effects on capillary-forming EC themselves. Live/dead staining of the tube assays after different time points could be helpful to further characterize the viability of single cells influenced by damaging effects as such. In future studies, it should also be investigated how DMOG treatment affects ASC functions, such as matrix synthesis, as this might also have effects on endothelial tube formation support.

As we assumed that NPSNPs loaded with DMOG might offer a stronger hypoxic environment in their close proximity, which might be harmful to the cells, only 50 µM DMOG was delivered to the hydrogels. Here, we did observe significantly higher values for relative tube area, number of junctions as well as total vessel length for wells treated with DMOG compared to wells of the control on day 4 of the culture. This could indicate a supportive effect of DMOG on NPSNPs for endothelial tube formation most likely by an enhanced VEGF production of ASC acting directly on tube forming EC (Figs 6 and 7). This effect, however, should be considered as transient, as no significant differences could be observed between the groups for either tube parameter on day 7 of the assay.

Although we have shown in a previous study [47] that endothelial cells from different origins behave very similar in terms of tube formation capabilities and tube parameters, future studies should be performed in order to determine the possible effect of DMOG on endothelial cells from specialized vascular beds, especially when generating tissue-specific constructs in the future.

It could be assumed that already NPSNPs without DMOG loading may affect endothelial tube formation, i.e. by changing the gel stiffness or by the influence of released silica species on the cells. Thus, the same tube parameters as mentioned above were analysed also for cells supplied with unloaded NPSNPs. In the presence of unloaded NPSNPs, values for all examined parameters were significantly lower compared to cells treated with 50 µM DMOG and the control. This again underlines the beneficial effects of DMOG-loaded NPSNPs on tube formation, which should be useful for tissue engineering approaches generating pre-vascularized tissue constructs. In this context, it is noteworthy that even a relatively short time frame of 4 days, in which increased tube formation was observed, could be highly beneficial for *in vivo* applications. Several studies applying different types of tissue constructs have shown inosculation of their implants to happen within the first 2–8 days after implantation [48–52]. Laschke *et al*. have stated that the state of maturation of the pre-formed capillary network in the tissue construct plays an important role for the success of inosculation and a rather rapid perfusion of the construct is favourable, which is also supported by Shepherd *et al*. [53, 54]. In their study, Laschke *et al*. [54, 55] have found that inosculation needs the pre-formed capillary network within the tissue construct to form new sprouts when already implanted in the host, which can then connect to the host vasculature. This is most likely not given in tissue constructs that were pre-vascularized for longer time periods.

## Summary and outlook

In this study, we showed that DMOG release from NPSNPs is a very promising strategy in order to support endothelial tube formation. We have clearly demonstrated supporting effects of NPSNPs loaded with DMOG in a co-culture of RFP-HUVEC and ASC in vascular tube formation assays. Moreover, we demonstrated upregulation of VEGF expression in ASC upon DMOG treatment. Nonetheless, further experiments are needed in the future to determine how the hypoxic gradient in a hydrogel can be designed, as we have seen that a DMOG concentration as low as 100 µM could have inhibiting effects on endothelial tube formation.

For future experiments, it is also of great interest to evaluate the effect of *in vitro* perfusion in combination with chemically induced hypoxia on endothelial tube formation in fibrin hydrogels. NPSNPs with their high adaptability to various delivery demands can be further developed to fit different release strategies.

## Supplementary data

Supplementary data are available at *REGBIO* online.

## Supplementary Material

rbab039_Supplementary_Data

## References

[1] Kannan RY, Salacinski HJ, Sales K et al The roles of tissue engineering and vascularisation in the development of micro-vascular networks: a review. Biomaterials 2005;26:1857–75. 15576160 10.1016/j.biomaterials.2004.07.006

[2] Ma B, Li M, Fuchs S et al Short-term hypoxia promotes vascularization in co-culture system consisting of primary human osteoblasts and outgrowth endothelial cells. J Biomed Mater Res A 2020;108:7–18. 31430039 10.1002/jbm.a.36786

[3] Befani C, Liakos P. Hypoxia upregulates integrin gene expression in microvascular endothelial cells and promotes their migration and capillary-like tube formation. Cell Biol Int 2017;41:769–78. 28418174 10.1002/cbin.10777

[4] Marchbank T, Mahmood A, Harten S et al Dimethyloxalyglycine stimulates the early stages of gastrointestinal repair processes through VEGF-dependent mechanisms. Lab Invest 2011;91:1684–94. 21876537 10.1038/labinvest.2011.129

[5] Zhou M, Hou J, Li Y et al The pro-angiogenic role of hypoxia inducible factor stabilizer FG-4592 and its application in an in vivo tissue engineering chamber model. Sci Rep 2019;9:6035. 30988335 10.1038/s41598-019-41924-5 PMC6465281

[6] Zamora DO, Natesan S, Becerra S et al Enhanced wound vascularization using a dsASCs seeded FPEG scaffold. Angiogenesis 2013;16:745–57. 23709171 10.1007/s10456-013-9352-y

[7] Natesan S, Zhang G, Baer DG et al A bilayer construct controls adipose-derived stem cell differentiation into endothelial cells and pericytes without growth factor stimulation. Tissue Eng Part A 2011;17:941–53. 21083419 10.1089/ten.tea.2010.0294 PMC3117235

[8] Nelson BS, Kremer DM, Lyssiotis CA. New tricks for an old drug. Nat Chem Biol 2018;14:990–1. 30297874 10.1038/s41589-018-0137-x

[9] Fullriede H, Abendroth P, Ehlert N et al PH-responsive release of chlorhexidine from modified nanoporous silica nanoparticles for dental applications. BioNanoMaterials 2016;17:59–72.

[10] Yasmin R, Rao S, Bremmell KE et al Porous silica-supported solid lipid particles for enhanced solubilization of poorly soluble drugs. AAPS J 2016;18:876–85. 27048207 10.1208/s12248-015-9864-z

[11] Chen F, Hableel G, Zhao ER et al Multifunctional nanomedicine with silica: role of silica in nanoparticles for theranostic, imaging, and drug monitoring. J Colloid Interface Sci 2018;521:261–79. 29510868 10.1016/j.jcis.2018.02.053 PMC5899957

[12] Morin KT, Tranquillo RT. In vitro models of angiogenesis and vasculogenesis in fibrin gel. Exp Cell Res 2013;319:2409–17. 23800466 10.1016/j.yexcr.2013.06.006 PMC3919069

[13] Linnes MP, Ratner BD, Giachelli CM. A fibrinogen-based precision microporous scaffold for tissue engineering. Biomaterials 2007;28:5298–306. 17765302 10.1016/j.biomaterials.2007.08.020 PMC2140252

[14] Chung Y Il, Ahn KM, Jeon SH et al Enhanced bone regeneration with BMP-2 loaded functional nanoparticle-hydrogel complex. J Control Release 2007;121:91–9. 17604871 10.1016/j.jconrel.2007.05.029

[15] Ye Q, Zünd G, Benedikt P et al Fibrin gel as a three dimensional matrix in cardiovascular tissue engineering. Eur J Cardio-Thoracic Surg 2000;17:587–91. 10.1016/s1010-7940(00)00373-0 10814924

[16] Cho SW, Kim I, Kim SH et al Enhancement of adipose tissue formation by implantation of adipogenic-differentiated preadipocytes. Biochem Biophys Res Commun 2006;345:588–94. 16690020 10.1016/j.bbrc.2006.04.089

[17] Hojo M, Inokuchi S, Kidokoro M et al Induction of vascular endothelial growth factor by fibrin as a dermal substrate for cultured skin substitute. Plast Reconstr Surg 2003;111:1638–45. 12655209 10.1097/01.PRS.0000053842.90564.26

[18] Lau S, Schrimpf C, Klingenberg M et al Evaluation of autologous tissue sources for the isolation of endothelial cells and adipose tissue-derived mesenchymal stem cells to pre-vascularize tissue-engineered vascular grafts. BioNanoMaterials 2015;16:309–21.

[19] Qiao ZA, Zhang L, Guo M et al Synthesis of mesoporous silica nanoparticles via controlled hydrolysis and condensation of silicon alkoxide. Chem Mater 2009;21:3823–9.

[20] Schmidt N, Schulze J, Warwas DP et al Long-term delivery of brain-derived neurotrophic factor (BDNF) from nanoporous silica nanoparticles improves the survival of spiral ganglion neurons in vitro. PLoS One 2018;13:e0194778. 29584754 10.1371/journal.pone.0194778 PMC5870973

[21] Zudaire E, Gambardella L, Kurcz C et al A computational tool for quantitative analysis of vascular networks. PLoS One 2011;6:e27385. 22110636 10.1371/journal.pone.0027385 PMC3217985

[22] Novosel EC, Kleinhans C, Kluger PJ. Vascularization is the key challenge in tissue engineering. Adv Drug Deliv Rev 2011;63:300–11. 21396416 10.1016/j.addr.2011.03.004

[23] Rivron NC, Liu J, Rouwkema J et al Engineering vascularised tissues in vitro. eCM 2008;15:27–40. 18288631 10.22203/ecm.v015a03

[24] Pill K, Hofmann S, Redl H et al Vascularization mediated by mesenchymal stem cells from bone marrow and adipose tissue: a comparison. Cell Regen 2015;4:8. 26500761 10.1186/s13619-015-0025-8 PMC4619361

[25] Verseijden F, Posthumus-Van Sluijs SJ, Pavljasevic P et al Adult human bone marrow-and adipose tissue-derived stromal cells support the formation of prevascular-like structures from endothelial cells in vitro. Tissue Eng Part A 2010;16:101–14. 19642855 10.1089/ten.TEA.2009.0106

[26] Rohringer S, Hofbauer P, Schneider KH et al Mechanisms of vasculogenesis in 3D fibrin matrices mediated by the interaction of adipose-derived stem cells and endothelial cells. Angiogenesis 2014;17:921–33. 25086616 10.1007/s10456-014-9439-0

[27] Choi JR, Yong KW, Wan Safwani WKZ. Effect of hypoxia on human adipose-derived mesenchymal stem cells and its potential clinical applications. Cell Mol Life Sci 2017;74:2587–600. 28224204 10.1007/s00018-017-2484-2 PMC11107561

[28] Choi JR, Pingguan-Murphy B, Abas WABW et al In situ normoxia enhances survival and proliferation rate of human adipose tissue-derived stromal cells without increasing the risk of tumourigenesis. PLoS One 2015;10:e0115034. 25615717 10.1371/journal.pone.0115034 PMC4304807

[29] Fets L, Driscoll PC, Grimm F et al MCT2 mediates concentration-dependent inhibition of glutamine metabolism by MOG. Nat Chem Biol 2018;14:1032–42. 30297875 10.1038/s41589-018-0136-y PMC6298574

[30] Chen C, Tang Q, Zhang Y et al Metabolic reprogramming by HIF-1 activation enhances survivability of human adipose-derived stem cells in ischaemic microenvironments. Cell Prolif 2017;50:e12363. 10.1111/cpr.12363 PMC6529110 28752896

[31] Ding H, Gao YS, Wang Y et al Dimethyloxaloylglycine increases the bone healing capacity of adipose-derived stem cells by promoting osteogenic differentiation and angiogenic potential. Stem Cells Dev 2014;23:990–1000. 24328551 10.1089/scd.2013.0486 PMC3996975

[32] Zhang J, Feng Z, Wei J et al Repair of critical-sized mandible defects in aged rat using hypoxia preconditioned BMSCs with up-regulation of Hif-1α. Int J Biol Sci 2018;14:449–60. 29725266 10.7150/ijbs.24158 PMC5930477

[33] Milkiewicz M, Pugh CW, Egginton S. Inhibition of endogenous HIF inactivation induces angiogenesis in ischaemic skeletal muscles of mice. J Physiol 2004;560:21–6. 15319416 10.1113/jphysiol.2004.069757 PMC1665195

[34] Zhu T, Park HC, Son KM et al Effects of dimethyloxalylglycine on wound healing of palatal mucosa in a rat model. BMC Oral Health 2015;15:60. 25981588 10.1186/s12903-015-0047-1 PMC4434535

[35] Rendina AR, Pietrak B, Smallwood A et al Mutant IDH1 enhances the production of 2-hydroxyglutarate due to its kinetic mechanism. Biochemistry 2013;52:4563–77. 23731180 10.1021/bi400514k

[36] He Q, Zhang Z, Gao Y et al Intracellular localization and cytotoxicity of spherical mesoporous silica nano-and microparticles. Small 2009;5:2722–9. 19780070 10.1002/smll.200900923

[37] Asefa T, Tao Z. Biocompatibility of mesoporous silica nanoparticles. Chem Res Toxicol 2012;25:2265–84. 22823891 10.1021/tx300166u

[38] Ha SW, Sikorski JA, Weitzmann MN et al Bio-active engineered 50 nm silica nanoparticles with bone anabolic activity: therapeutic index, effective concentration, and cytotoxicity profile in vitro. Toxicol In Vitro 2014;28:354–64. 24333519 10.1016/j.tiv.2013.12.001 PMC3926416

[39] Bauer AT, Strozyk EA, Gorzelanny C et al Cytotoxicity of silica nanoparticles through exocytosis of von Willebrand factor and necrotic cell death in primary human endothelial cells. Biomaterials 2011;32:8385–93. 21840590 10.1016/j.biomaterials.2011.07.078

[40] Williams S, Neumann A, Bremer I et al Nanoporous silica nanoparticles as biomaterials: evaluation of different strategies for the functionalization with polysialic acid by step-by-step cytocompatibility testing. J Mater Sci Mater Med 2015;26:125. 25690616 10.1007/s10856-015-5409-3

[41] Neumann A, Christel A, Kasper C et al BMP2-loaded nanoporous silica nanoparticles promote osteogenic differentiation of human mesenchymal stem cells. RSC Adv 2013;3:24222–30.

[42] Kim IY, Joachim E, Choi H et al Toxicity of silica nanoparticles depends on size, dose, and cell type. Nanomedicine 2015;11:1407–16. 25819884 10.1016/j.nano.2015.03.004

[43] Yang X, Li Y, Liu X et al The stimulatory effect of silica nanoparticles on osteogenic differentiation of human mesenchymal stem cells. Biomed Mater 2017;12:015001. 10.1088/1748-605X/12/1/015001 27910816

[44] Esser TU, Roshanbinfar K, Engel FB. Promoting vascularization for tissue engineering constructs: current strategies focusing on HIF-regulating scaffolds. Expert Opin Biol Ther 2019;19:105–118. 30570406 10.1080/14712598.2019.1561855

[45] Agis H, Watzek G, Gruber R. Prolyl hydroxylase inhibitors increase the production of vascular endothelial growth factor by periodontal fibroblasts. J Periodontal Res 2012;47:165–73. 21954882 10.1111/j.1600-0765.2011.01415.x

[46] Hellström M, Gerhardt H, Kalén M et al Lack of pericytes leads to endothelial hyperplasia and abnormal vascular morphogenesis. J Cell Biol 2001;153:543–54. 11331305 10.1083/jcb.153.3.543 PMC2190573

[47] Lau S, Eicke D, Oliveira MC et al Low immunogenic endothelial cells maintain morphological and functional properties required for vascular tissue engineering. Tissue Eng Part A 2018;24:432–47. 28978275 10.1089/ten.TEA.2016.0541

[48] Ohmori S, Kurata K. Experimental studies on the blood supply to various types op skin grafts in rabbits using isotope p32. Plast Reconstr Surg 1960;25:547–55. 10.1097/00006534-196006000-00001 14428467

[49] Lindenblatt N, Calcagni M, Contaldo C et al A new model for studying the revascularization of skin grafts in vivo: the role of angiogenesis. Plast Reconstr Surg 2008;122:1669–80. 19050519 10.1097/PRS.0b013e31818cbeb1

[50] Best TJ, Mackinnon SE, Midha R et al Revascularization of peripheral nerve autografts and allografts. Plast Reconstr Surg 1999;104:152–60. 10597688

[51] Chalfoun C, Scholz T, Cole MD et al Primary nerve grafting: a study of revascularization. Microsurgery 2003;23:60–5. 12616521 10.1002/micr.10082

[52] Albrektsson T. In vivo studies of bone grafts: the possibility of vascular anastomoses in healing bone. Acta Orthop Scand 1980;51:9–17. 6990682 10.3109/17453678008990763

[53] Shepherd BR, Chen HYS, Smith CM et al Rapid perfusion and network remodeling in a microvascular construct after implantation. Arterioscler Thromb Vasc Biol 2004;24:898–904. 14988090 10.1161/01.ATV.0000124103.86943.1e

[54] Laschke MW, Mussawy H, Schuler S et al Short-term cultivation of in situ prevascularized tissue constructs accelerates inosculation of their preformed microvascular networks after implantation into the host tissue. Tissue Eng Part A 2011;17:841–53. 20973748 10.1089/ten.TEA.2010.0329

[55] Laschke MW, Vollmar B, Menger MD. Inosculation: connecting the life-sustaining pipelines. Tissue Eng Part B Rev 2009;15:455–65. 19552605 10.1089/ten.TEB.2009.0252

