# Supplementary material for: Chemically induced hypoxia by dimethyloxalylglycine (DMOG)-loaded nanoporous silica nanoparticles supports endothelial tube formation by sustained VEGF release from adipose tissue-derived stem cells
Source: Regen Biomater. 2021 Aug 14;8(5):rbab039. doi: 10.1093/rb/rbab039 (PMC8363767; doi:10.1093/rb/rbab039)
Supplement: rbab039_Supplementary_Data [file rbab039_supplementary_data.zip › Supplementary information.docx]

**Supplementary information**

Here, some supplementary information about the characterization of the unmodified and amino-modified nanoporous silica nanoparticles (NPSNPs) is presented. Transmission electron microscopy images of the unmodified NPSNPs show their monodisperse character and an average diameter of about 45 nm (see Supplementary Figure 1A, Supp. Fig. 1A). After the amino modification these properties are maintained as depicted in Supp. Fig. 1B. PH-dependent zeta-potential measurements prove the successful attachment of amino groups by strong positive zeta potential values in the lower pH range (Supp. Fig. 1C). This positive charge results from the protonation of amino groups on the surface. The unmodified nanoparticles for comparison show a negative zeta potential up to a pH value of 4. The isoelectric point is shifted from about 4 for the unmodified to 7.4 for the amino-modified NPSNPs. A further hint for a successful amino-modification of the NPSNPs can be found in the results of the nitrogen sorption investigations (Supp. Fig. 1D). The BET surface area calculated from the isotherms decreases from a value of about 950 m^2^ g^-1^ for the unmodified NPSNPs to about 330 m^2^ g^-1^ for the amino-modified NPSNPs (concerning pore volume from 1.0 cm^3^ g^-1^ to 0.4 cm^3^ g^-1^) which shows that a high nanoporosity is still present. This reduction of surface area and pore volume while the average pore size of 3 nm for both materials remains unaffected is caused by partial pore blocking which might result either from a slight dissolution/reprecipitation of the nanoparticles during the modification treatment or from the deposition of organosilane residues on the particle surface, which can be formed by self-condensation of the silanisation reagent in solution.


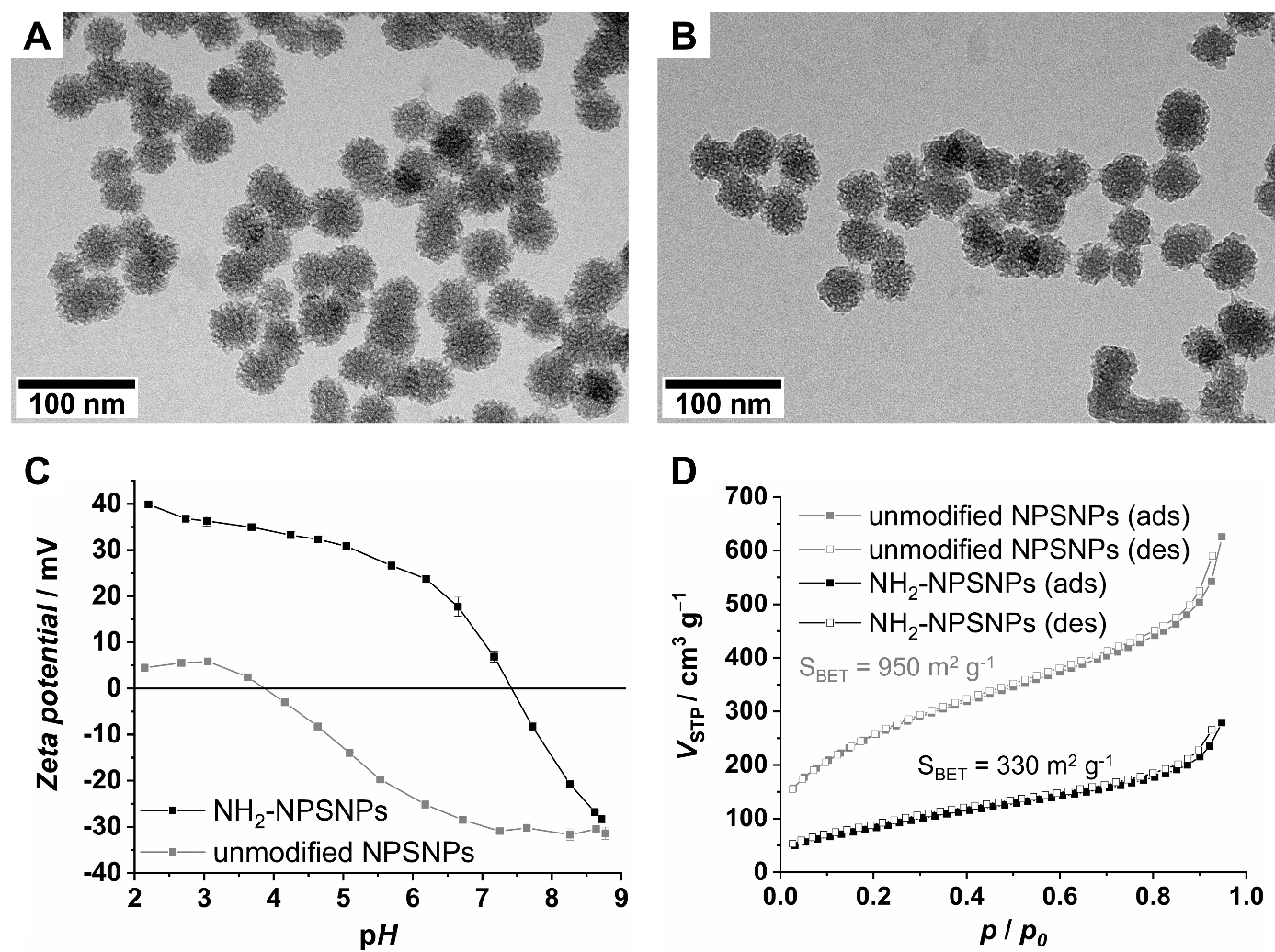


**Supp. Fig. 1: Characterization of amino-modified NPSNPs (NH_2_-NPSNPs) in comparison to unmodified NPSNPs.** Transmission electron microscope image of unmodified NPSNPs (A) and amino-modified NPSNPs (B); pH-dependent zeta-potential measurements of unmodified (grey) and amino-modified (black) NPSNPs (C); nitrogen physisorption isotherms of unmodified (grey) and amino-modified (black) NPSNPs (D) (ads: adsorption branch; des: desorption branch).
