# Supplementary figures and images for: Chemically induced hypoxia by dimethyloxalylglycine (DMOG)-loaded nanoporous silica nanoparticles supports endothelial tube formation by sustained VEGF release from adipose tissue-derived stem cells
Source: Regen Biomater. 2021 Aug 14;8(5):rbab039. doi: 10.1093/rb/rbab039 (PMC8363767; doi:10.1093/rb/rbab039)

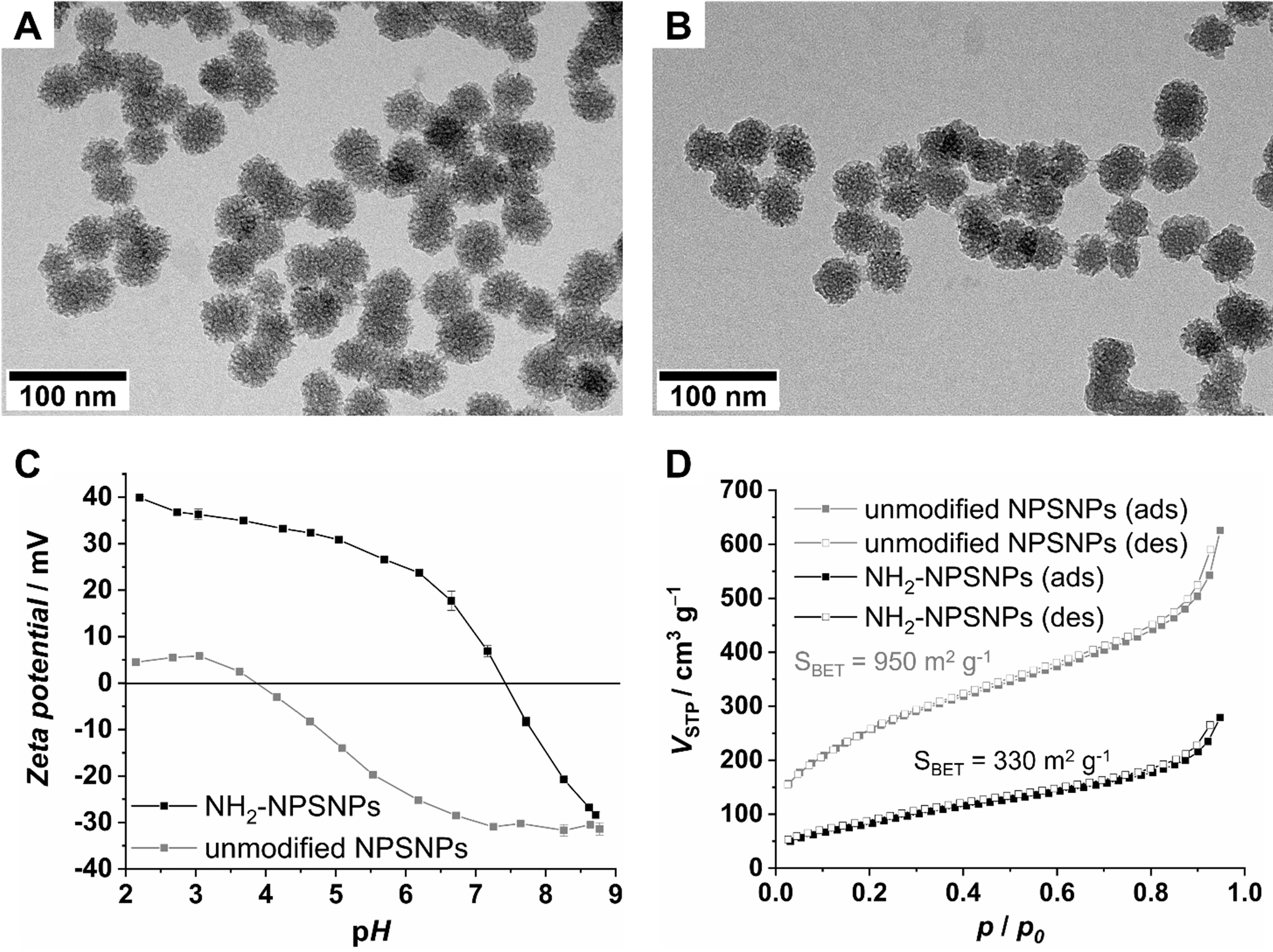

Supplement: rbab039_Supplementary_Data [file rbab039_supplementary_data.zip › Supp.Fig.1.tif]

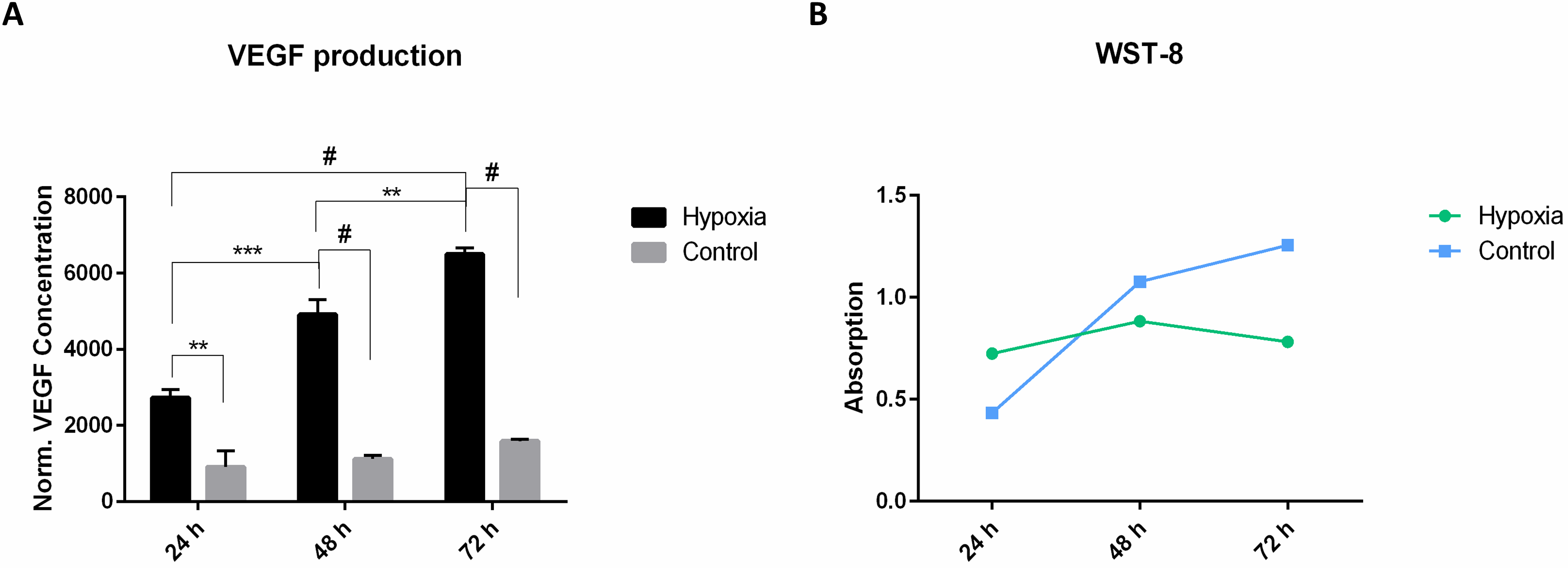

Supplement: rbab039_Supplementary_Data [file rbab039_supplementary_data.zip › Supp.Fig.2.tif]

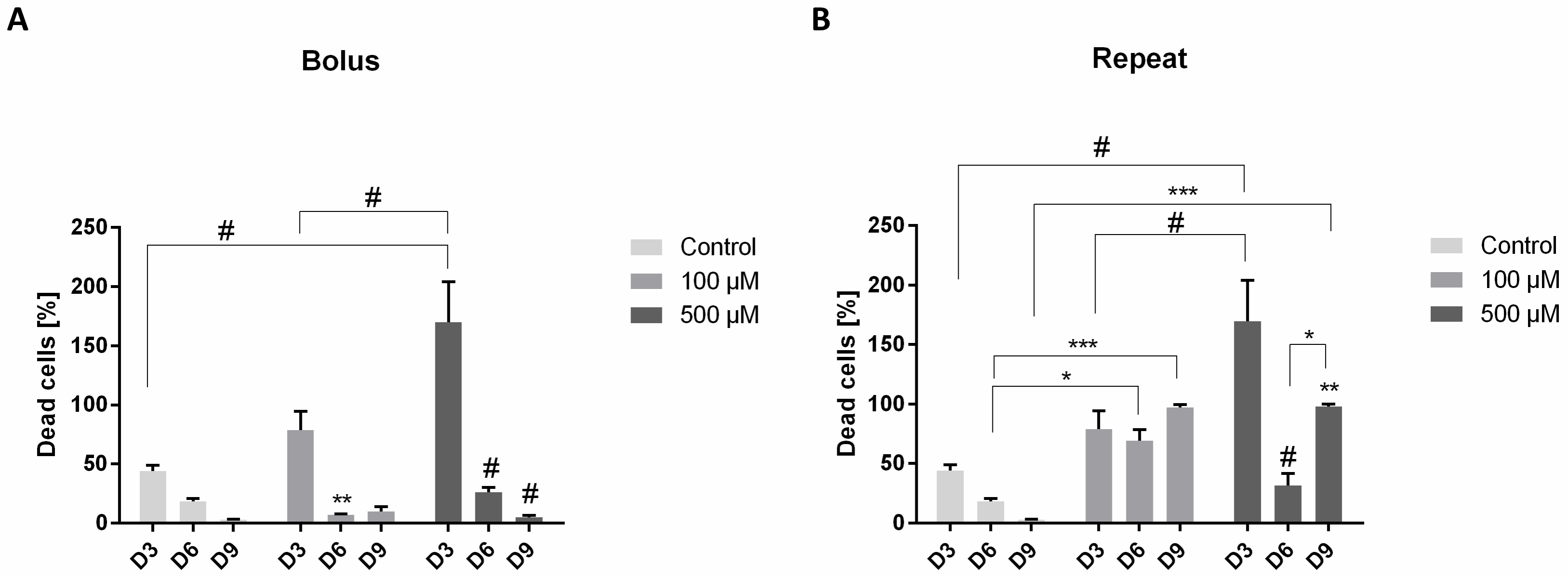

Supplement: rbab039_Supplementary_Data [file rbab039_supplementary_data.zip › Supp.Fig.3.tif]
